# Chronic sildenafil citrate use decreases retinal vascular endothelial growth factor expression in diabetic rats: a pilot study

**DOI:** 10.1186/s40942-023-00480-x

**Published:** 2023-07-17

**Authors:** Osama A. Sorour, Elsayed Nassar, Naglaa Sarhan, Noha El-Anwar, Reem A ElKholy, Dina M. Tahoon, Aalaa Sweilam, Dina Tadros

**Affiliations:** 1 Department of Ophthalmology, Faculty of Medicine, Tanta University, Tanta, Egypt; 2 Department of Histology, Faculty of Medicine, Tanta University, Tanta, Egypt; 3 Department of Pathology, Faculty of Medicine, Tanta University, Tanta, Egypt; 4 Department of Pathology, Armed Forces College of Medicine, Heliopolis, Egypt; 5 Department of Pharmacology, Faculty of Medicine, Tanta University, Tanta, Egypt; 6 Department of Pharmacology, School of medicine, Badr University, Badr, Egypt; 7 Department of Clinical Pathology, Faculty of Medicine, Tanta University, Tanta, Egypt

**Keywords:** VEGF, Sildenafil, PDEI-5, Diabetic retinopathy, ELISA, Immunohistochemistry

## Abstract

**Background:**

Sildenafil citrate (SC) attenuates endothelial dysfunction. However, its effects on diabetic retinopathy (DR), which is mainly a microvascular disease, remain unclear. Vascular endothelial growth factor (VEGF) is known to be a critical mediator of DR. Therefore, we investigated the effects of SC on diabetic retina by measuring VEGF levels.

**Methods:**

In this study, twenty-eight rats were divided into the following groups: group I, the control group; group II, rats with streptozotocin-induced diabetes; group III, rats with streptozotocin-induced diabetes receiving daily oral sildenafil at 1 mg/kg; and group IV, rats with streptozotocin-induced diabetes receiving high-dose daily sildenafil at 2.5 mg/kg. After 3 months, VEGF was measured in the retina specimen in one eye and the vitreous body in the other eye by immunohistochemistry and enzyme-linked immunosorbent assay, respectively.

**Results:**

We found that VEGF expression in the retina was low in all rats from groups I and IV and in 30% of rats from group III; 80% of rats in group II demonstrated high VEGF expression in the retinae (P < 0.001). VEGF concentrations in the vitreous body samples were 32 ± 2, 61 ± 4, 44 ± 5, and 36 ± 3 pg/l in groups I–IV, respectively (P < 0.001).

**Conclusion:**

VEGF decreased significantly in the eyes of diabetic rats after chronic oral sildenafil citrate treatment. SC may have a modifying/attenuating effect on DR. However, further studies are needed to evaluate its use as an adjunctive treatment.

## Background

Sildenafil citrate (SC), which is a selective phosphodiesterase inhibitor, has been associated with increased availability of cGMP and nitric oxide (NO) in tissues, producing a prominent vasodilatory effect. In addition to its widespread use for the treatment of erectile dysfunction and pulmonary arterial hypertension, sildenafil citrate (SC) is used off-label for benign prostate hypertrophy, Raynaud’s phenomenon and the prevention and treatment of high-altitude pulmonary oedema. SC actions are mainly elicited through phosphodiesterase-5 (PDE-5), which is widely distributed in vascular smooth muscle, corpus cavernosum, lungs and platelets. However, to a lesser degree, SC can also affect PDE-6 (nearly 10% of the activity of PDE-5), which is present only in photoreceptors. PDE-6 is known to play an important role in the phototransduction cascade. Therefore, the visual side effects of SC may be related to the inhibition of PDE-6 [1]. Most described visual side effects included subtle changes in vision in the form of blue/green color deficits, temporary blurring, and enhanced brightness in 2–3% of the patients using SC, due to its effect on PDE6- mediated phototransduction process in the photoreceptor outer segments [2].

Diabetes mellitus (DM) is associated with endothelial dysfunction, reducing NO-dependent vasodilation and increasing the production of pro-inflammatory factors, resulting in microvascular diseases in the retina, kidney and other tissues [3]. Diabetic retinopathy (DR), which is a common sequela of DM, has been identified as a leading cause of vision loss. The first stage of DR is non-proliferative diabetic retinopathy, which is characterized by prevalent microaneurysms and retinal hemorrhages. The second stage, proliferative diabetic retinopathy, is caused by progressive non-perfusion of capillaries leading to neovascularization. Neovascularization and excessive vascular permeability in the retina are linked to the upregulation of vascular endothelial growth factor (VEGF) [4].

Chronic administration of SC may improve endothelial dysfunction, due to the enhancement of NO/cGMP signaling and antioxidant activity via inhibition of NADPH oxidase activity and reduction of superoxide formation [5]. Accordingly, PDE5 inhibitors have been tested for the prevention/treatment of various vascular disorders, including diabetic vasculopathy [6], retinal ischemia/reperfusion injury [7], diabetic nephropathy [8] and Alzheimer’s disease [9].

PDE-5 is present in choroidal and retinal blood vessels, and SC is known to increase ocular blood flow, especially choroidal blood flow [10], most probably through NO-mediated vasodilatation [11, 12]. In addition, NO may have inhibitory effects on retinal vascular obliteration and subsequent proliferative retinopathies [13]. Based on the increased ocular blood flow, PDE-5 inhibitors may affect diseases involving increased choroidal thickness, such as central serous chorioretinopathy, and may be a therapeutic option for other diseases caused by choroidal and/or retinal ischemia [14].

Despite the theoretical benefits, the effects of SC on DR have not been extensively investigated. Thus, in this study, we aimed to investigate the effects of sildenafil on the retina and vitreous body in rats with DM versus healthy rats by observing the changes in VEGF.

## Materials and methods

This study was conducted at the Faculty of Medicine, Tanta University, Egypt, from September to December 2021. Male Sprague–Dawley (SD) rats weighing 120–150 g were maintained under controlled temperature (23 ± 2 °C), humidity (50%) and lighting (12 h light/12 h dark) in the Pharmacology Department Animal House. The rats were fed a standard laboratory diet and given free access to tap water. The age range of rats at the beginning of the experiment was 10–12 weeks. All animals received humane care in compliance with the institutional animal care guidelines and with adherence to National Research Council’s Guide for the Care and Use of Laboratory Animals. The study protocol was approved by the Experimental Animal Ethical Committee of Tanta University.

The animals were divided into four groups (n = 7 per group). Group I (Control group) received saline intraperitoneally (IP) at day 0 and 0.5 ml of distilled water daily by oral gavage. Group II (Diabetic group) received a single IP injection of streptozotocin (STZ) at a dose of 65 mg/kg on day 0 to induce DM. Serum glucose concentrations were measured approximately 3 days after injection, and rats found to have a high glucose concentration (> 16 mmoL/L) were considered diabetic. DM was induced in group III (low-dose sildenafil diabetic group) as described above, and sildenafil 1 mg/kg was given daily by oral gavage for 3 months. DM was induced in group IV (high-dose sildenafil diabetic group), as described above, and sildenafil 2.5 mg/kg was given daily by oral gavage for 3 months. The animal equivalent dose (AED) was calculated from the minimum effective dose for erectile dysfunction, 25 mg, and considering the potential for prolonged treatment and the need for minimizing sexual & possible adverse effects of the drug, the experimental dosing were selected around this AED [15].

Sildenafil was prepared daily by crushing Viagra (Sildenafil citrate) 100 mg tablets (Pfizer INC; USA) into powder and dissolving the powder in distilled water to final concentrations of 1 mg/kg and 2.5 mg /kg. At the end of the experiment, the rats were anaesthetized, and their eyes were then removed. One globe from each rat was selected randomly for immunohistochemistry, while the other was used for enzyme-linked immunosorbent assay (ELISA) evaluation of VEGF. After the removal of the globes, animals were killed immediately.

### Immunohistochemical staining for VEGF

Retinae were dissected from the globe and immediately immersed in a 10% formaldehyde solution for fixation. The specimens were processed for the preparation of paraffin blocks. A sledge microtome was used to produce 4 μm sections from the paraffin blocks. For immunohistochemical staining, sections were applied to glass slides coated with 2% saline solution (APES; cat. no.: A3648 Sigma, Aldrich). Before staining, the slides were baked at 36 °C for 24 h, deparaffinized and rehydrated. The monoclonal anti-VEGF mouse antibody (clone VG1, Dako, USA) was used for immunohistochemical staining. VEGF expression was scored according to the intensity of the staining reaction as negative (−), weak (+), medium (++) and strong (+++) and subsequently categorized into low and high expression grades. The low expression grade included −/+, whereas the high included ++/+++ staining [16]. The grading of immunohistochemical staining was performed independently by two expert researchers; NS & NE, and any discrepancy was resolved by open discussion.

### VEGF ELISA

The vitreous body, not the retinal tissue, was analysed. Careful dissection of the globe was performed to obtain the vitreous body without other tissue or blood contamination. Vitreous samples were homogenised in phosphate-buffered saline (pH 7.4). The homogenate was centrifuged at 3000 rpm for 20 min, and the supernatant was aliquoted and kept at -20 °C until use. Vitreous VEGF-A levels were evaluated using a rat sandwich ELISA kit (Shanghai Sunred Biological Technology Co., Ltd, China) according to the manufacturer’s instructions. The micro-ELISA plate was pre-coated with anti-rat antibodies for VEGF-A. Standards and samples were added to the plate and combined with the specific antibody. Then, a biotinylated detection antibody and avidin-conjugated to horseradish peroxidase (HRP) were added successively to each microplate well and incubated. The substrate solution was added to each well, and blue colour developed. The stop solution turned into yellow in colour. The optical density was measured spectrophotometrically at a wavelength of 450 nm. Vitreous samples that were contaminated with blood or other tissues or of insufficient amount were discarded. This leads to inclusion of 7 rats in each group instead of the initial 10 rats at beginning of the experiment. However, the retinal specimens of all 10 rats were available for immunohistochemistry analysis.

### Statistical analysis

Quantitative data were expressed as mean (SD). Groups were compared using Chi-square and non-parametric Kruskal-Wallis tests for immunohistochemical data and ELISA measurements. Mann-Whitney U test was used for two groups ELISA-VEGF level comparison. A p-value less than 0.05 was considered significant.

## RESULTS

**Fig. 1:**
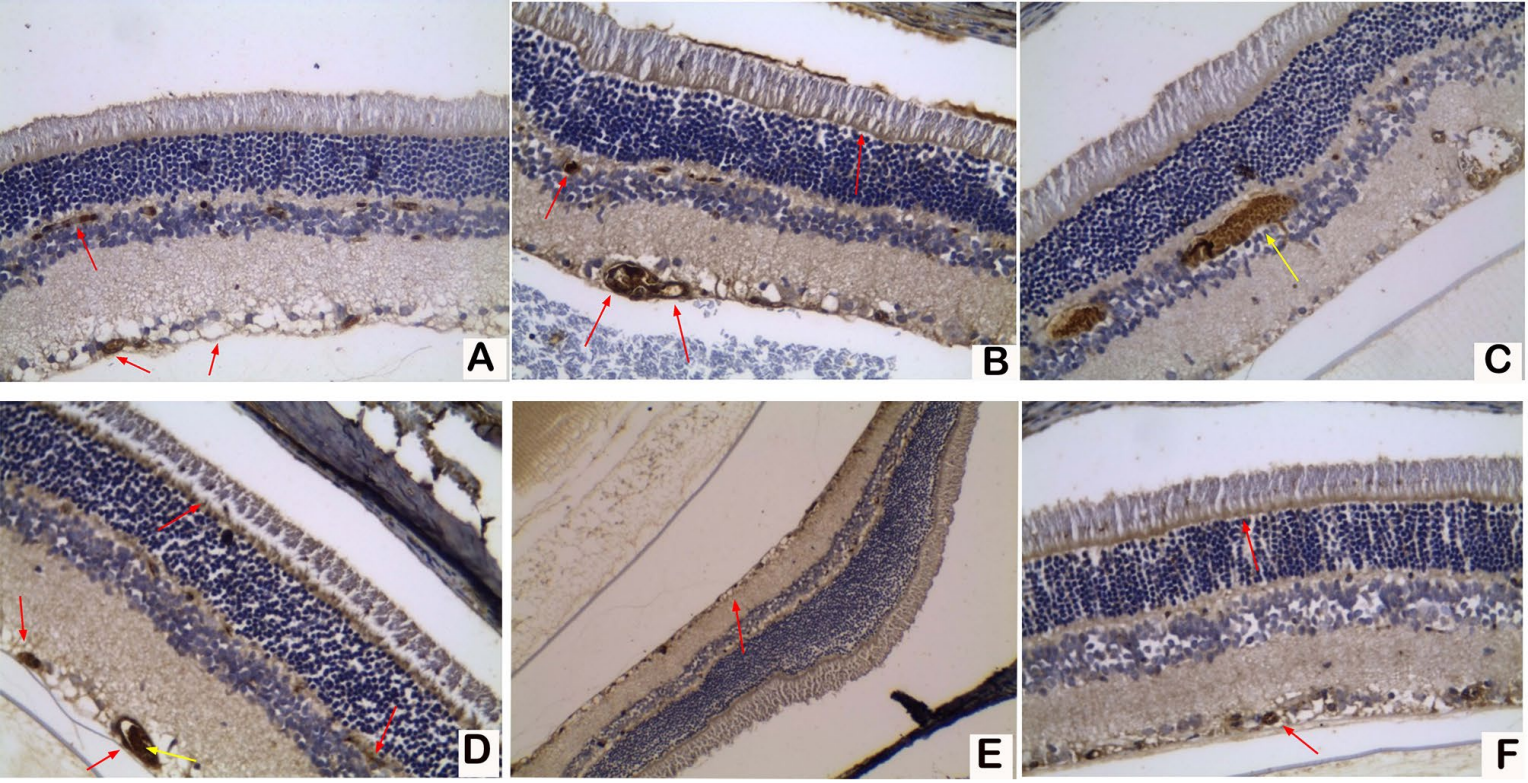
**VEGF expression in retinal tissues measured by immunohistochemistry** Red arrows refers to the VEGF immunohistochemical staining in the wall of endothelial cells. (panel A = Group I) exhibited low VEGF expression (x400). (B and C; Group II) exhibited high VEGF expression with high immunoreactivity in the ganglion cell layer (GCL), the inner nuclear layer (INL), and the border between the outer nuclear layer and the outer segment of the photoreceptors, and congestion of capillaries (yellow arrow) in the INL and GCL (x400). (D; Group III) exhibited high VEGF expression and capillary congestion (yellow arrow) (x400). (E and F; Group IV) exhibited diffuse low expression in two rats on low (x200) and high power (x400) views, respectively

According to the immunohistochemistry of retinal specimens, VEGF expression was low in all rats of the control group (group I) (Fig. 1A), while group II (diabetic group) exhibited high VEGF expression in 80% of rats, with a high degree of immunoreactivity in the ganglion cell layer (GCL), the inner nuclear layer (INL) and the border between the outer nuclear layer and the outer segment of photoreceptors. The retinae in group II also exhibited congestion of the capillaries in the INL and GCL (Fig. 1B and C). The expression of VEGF in group III (low-dose sildenafil diabetic group) was low in only 30% of rats (Fig. 1D) and was not significantly different from group II. However, 100% of retinae in group IV (high-dose sildenafil diabetic group) exhibited low VEGF expression (Fig. 1E and F), which was significantly lower compared with VEGF expression in group II (P < 0.001). These results indicate that the higher dose of sildenafil was effective in lowering VEGF expression. The expression of VEGF in the four groups is presented in Table 1.

**Table 1:** VEGF expression analysis by immunohistochemistry in the retinal tissues

VEGF	Group I		Group II		Group III		Group IV	
	N	%	N	%	N	%	N*	%
**Low**	10	100	2	20	3	30	10	100
**High**	0	0	8	80	7	70	0	0
** × ** ^**2**^ **test**	40.267							
**P value**	0.001*							
GI & GII	G I & G III		G I & G IV		GII & G III		GII & G IV	GIII & G IV
0.001*	0.001*		1.0		0.606		0.001*	0.001

**Abbreviation**: **VEGF**: vascular endothelial growth factor, **N**: number, * indicates statistical significance

**Fig. 2:**
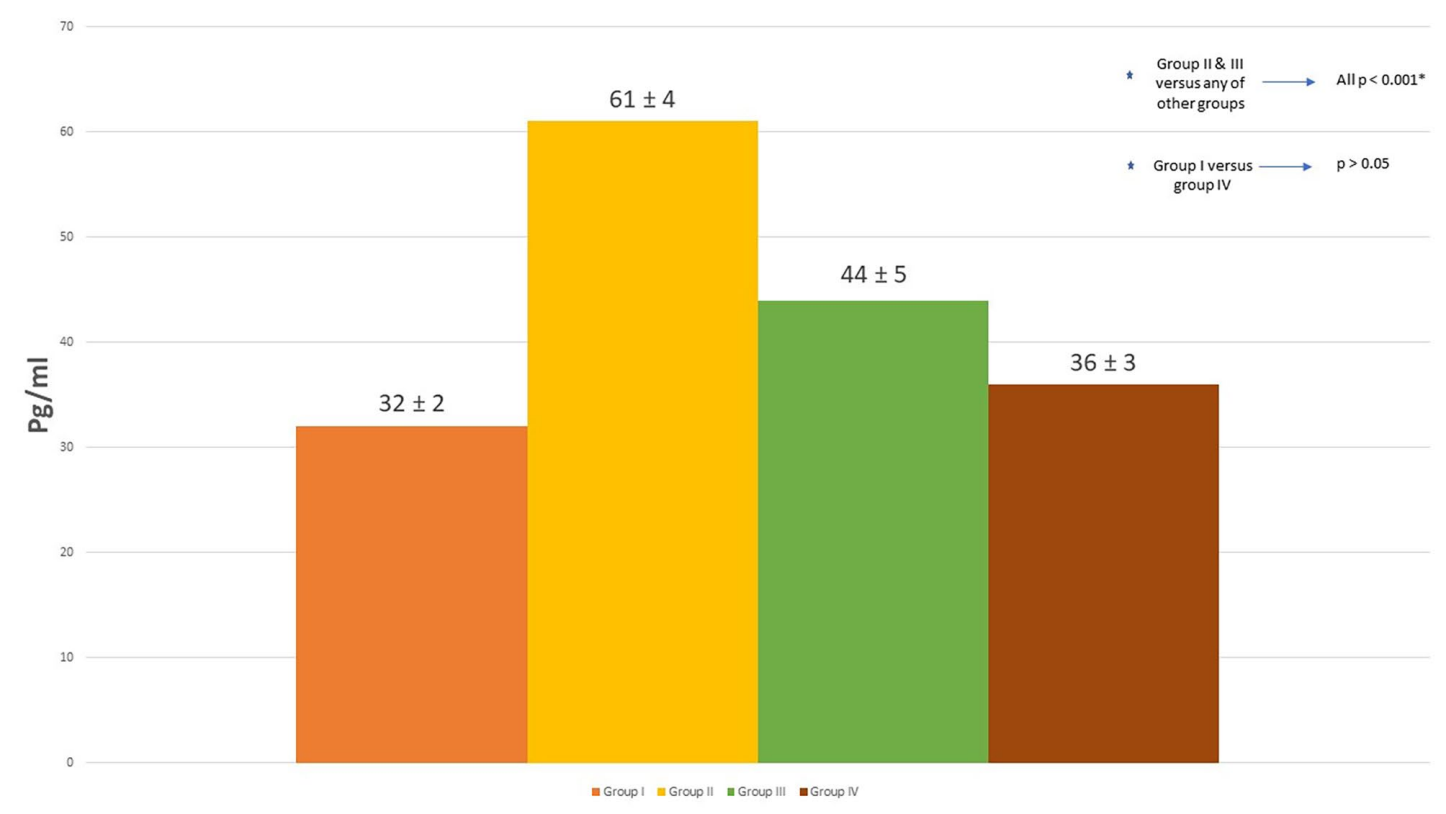
**VEGF levels in the vitreous body of the study groups by ELISA** This chart graph demonstrates the highest VEGF concentration observed in group II was significantly higher than all other groups. VEGF was significantly reduced in the rats with oral sildenafil. Furthermore, the reduction of VEGF by sildenafil was dose dependent with significantly lower concentration in rats using higher sildenafil dose reaching a level not significantly different from control group

The average (SD) VEGF-A levels in the vitreous bodies were 32 ± 2 pg/ml in group I, 61 ± 4 pg/ml in group II, 44 ± 5 pg/ml in group III and 36 ± 3 pg/ml in group IV (P < 0.001). Group-to-group analyses demonstrated significantly higher VEGF-A levels in group II compared to all other groups (P < 0.001). VEGF-A levels in group I were significantly lower than the levels in group III (p < 0.001), but not group IV. VEGF-A levels were also significantly higher in group III compared to that in group IV (p < 0.001) (Fig. 2).

## Discussion

Endothelial damage is known to be a main pathological feature of DR. Endothelial damage is caused by multiple interacting factors, including advanced glycosylation end products, pro-inflammatory cytokines, growth factors and oxidative stress. In addition, retinal microvasculopathy and neurodegeneration in diabetics result in the breakdown of the blood-retinal barrier (BRB), leading to oedema in the retinal structures, acellular capillaries, and neo-vessel formation [17].

VEGF is one of the most important mediators of DR development and progression. VEGF plays an important role in vascular permeability. VEGF also serves as a pro-inflammatory mediator and stimulator of angiogenesis and neovascularization, which are the main features of proliferative retinopathy. VEGF in the ocular fluid is associated with breakdown of the BRB [18]. In this study, chronic oral sildenafil was associated with low VEGF levels in the diabetic retina. This effect seemed to be dose-dependent, as VEGF was suppressed more in group IV compared with VEGF levels in group III. To the best of our knowledge, no other reports have demonstrated the effects of phosphodiesterase inhibitors in diabetic retinopathy or on VEGF levels in the diabetic retina. However, positive effects of phosphodiesterase inhibitors have been demonstrated in other diabetic tissues [19]. Our results suggest that suppression of detrimental VEGF expression by phosphodiesterase inhibitors may be a therapeutic option for improving DR. Giannetta et al. demonstrated that the direct action of chronic phosphodiesterase 5 inhibition on cardiac tissue has significantly improved diabetic cardiomyopathy [20].

NO induces VEGF synthesis under normoxic conditions; however, excessive NO may inhibit VEGF expression through an unidentified pathway [21]. Therefore, the effects of sildenafil treatment on VEGF tissue levels depend on the dosage and the organ. Several studies demonstrated conflicting effects of sildenafil on VEGF levels in different tissues, which may be related to the amount of NO produced [9, 22].

In our study we found that VEGF expression in the diabetic rats’ retinae was significantly low in the high dose SC group as compared to the rats the did not receive SC at all or received only small dose SC. Additionally, VEGF concentrations in the vitreous body samples were significantly elevated in the diabetic rats with no SC ingestion. However, rats that received high dose SC, demonstrated significantly low VEGF concentration that was comparable to the normal control group. Similar to our findings for VEGF suppression by SC, pentoxifylline, a non-selective phosphodiesterase inhibitor, significantly decreased VEGF in the renal tissue of diabetic rats at 4 and 8 weeks [8].

In addition to its known effects on NO, phosphodiesterase inhibitors may affect the diabetic retina by several other mechanisms. For example, various studies linked chronic phosphodiesterase 5 inhibition with enhanced insulin sensitivity and attenuated endothelial dysfunction [23, 24]. Furthermore, SC may prevent apoptosis after retinal ischemia [7]. In addition, sildenafil and tadalafil decreased pro-inflammatory cytokines in serum and tissues and inhibited oxidative and nitrosative stress in animal models [25].

In addition to the previous effects, SC has been associated with increased ocular blood flow, especially choroidal and, to a lesser extent, retinal blood flow [17]. Thus, SC may be a useful adjunct for treating ocular diseases that would benefit from increased choroidal blood flow. In humans, retinal blood flow decreases by approximately 33% in the early stages of diabetes but increases in the advanced stages correlating with the level of retinopathy. Hypothetically, preventing the early decrease in flow and ischemia may attenuate the hypoxia and angiogenesis that develop later [26]. Accordingly, SC may be used to increase ocular blood flow and prevent/slow DR. However, many studies have suggested that changes in ocular blood flow in response to SC are temporary and related to acute, not chronic, use [27]. Nevertheless, other studies suggest that SC can contribute to choroidal expansion, which may result from stromal rather than luminal components [1]. No studies describe the relationship between increased ocular blood flow and DR. However, Coleman et al. conducted a 2-year trial to evaluate the effect of sildenafil on age-related macular degeneration, wherein they concluded that a thickened Bruch’s membrane reduced the beneficial effects of increased perfusion, but the photoreceptor layer was maintained or improved, which may be due to PDE6 inhibition [28].

The use of SC for modulating DR may balance the possible visual side effects of the drug. Sildenafil is associated with complications, such as non-arteritic optic neuropathy, central serous retinopathy and increased intraocular pressure. However, randomised trials demonstrated modest, transient, and mainly colour perception changes in only 3–11% of users [29]. Furthermore, no conclusive evidence indicates a direct cause-effect relationship between PDE5 inhibition and vision-threatening ocular events. Moreover, men who use PDE5 inhibitors appear to suffer vision-threatening complications at the same frequency as the general population [30]. However, the chronic use of sildenafil citrate to treat diabetic retinopathy with the associated compromised perfusion requires further large-scale trials. Additionally, SC should not be used in nanophthalmos, pachychoroid, angle closure glaucoma, retinitis pigmentosa patients or carriers, eyes with small optic disc (prone for AION) [1, 31].

This study represents the first report in the literature on the effects of chronic SC treatment on DR and VEGF levels in the diabetic eye. However, our study also has several limitations, including the small number of rats and the limited duration of the experiment. Additionally, the grading of immunohistochemical staining was not blinded, however, double check of this grading by two experts gives us strong trust of these results. Finally, our studies focused on VEGF status only with SC use in diabetic rats, larger studies are required to investigate all the possible effects of SC on diabetic retinae.

In conclusion, we demonstrated a significant decrease in VEGF in the retina and vitreous body of diabetic rats after 3 months of oral SC. These findings indicate the potential adjunctive role of SC in improving/modifying diabetic retinopathy. However, additional large-scale long-term studies in the future are required to support our findings.

## Data Availability

The data that support the findings of this study are available from the authors upon reasonable request and after permission from Faculty of Medicine, Tanta University.
